# Physical activity and mobile phone addiction among Chinese college students: a chain mediation model of social anxiety and loneliness

**DOI:** 10.3389/fpsyg.2025.1545440

**Published:** 2025-04-22

**Authors:** Xueqiang Zhu, Haitao Niu, Qingying Zhu, Wenjia Chen

**Affiliations:** ^1^School of Competitive Sport, Shandong Sport University, Rizhao, China; ^2^School of Physical Education, Shandong University, Jinan, China; ^3^School of Physical Education, China University of Mining and Technology, Xuzhou, China

**Keywords:** physical activity, mobile phone addiction, social anxiety, loneliness, chain mediation, college students

## Abstract

**Introduction:**

Mobile phone addiction is increasingly prevalent among college students and can lead to various physical, psychological, and social problems. This study aimed to investigate the relationship between physical activity and mobile phone addiction among Chinese college students and to examine the potential chain mediating effects of social anxiety and loneliness in this relationship.

**Methods:**

A cross-sectional survey was conducted with 268 Chinese college students (62.7% male, mean age = 20.36 years, SD = 1.73) who completed standardized measures including the Physical Activity Rating Scale-3, Social Phobia Inventory, UCLA Loneliness Scale, and Mobile Phone Addiction Tendency Scale. Data were analyzed using Pearson’s correlations and PROCESS macro for mediation analyses with bootstrapping.

**Results:**

Physical activity had a significant negative effect on mobile phone addiction (*β* = −0.11, *p* < 0.05) with a total effect of *β* = −0.28 (*p* < 0.001). Social anxiety [*β* = −0.12, 95% CI (−0.18, −0.06)] and loneliness [*β* = −0.03, 95% CI (−0.06, −0.01)] independently mediated this relationship, while a significant chain mediation through both variables was also found [*β* = −0.02, 95% CI (−0.03, −0.01)], with all indirect effects accounting for 59.68% of the total effect.

**Discussion:**

These findings extend previous research by identifying the psychological mechanisms through which physical activity protects against mobile phone addiction, providing new theoretical insights into the sequential pathway from physical activity to reduced addiction, and practical implications for developing targeted interventions that combine physical activity promotion with strategies to reduce social anxiety and loneliness among college students.

## 1 Introduction

With the rapid development of information technology and the increasing prevalence of smartphones, mobile phone addiction has become a growing concern, particularly among college students (Sun et al., 2019). Mobile phone addiction, also known as problematic mobile phone use or smartphone addiction, is characterized by excessive or poorly controlled preoccupations, urges, or behaviors regarding mobile phone use that lead to impairment or distress (Lin et al., 2016). College students are especially vulnerable to mobile phone addiction due to their developmental stage, which is marked by increased independence, identity exploration, and the need for social connection (Arnett, 2000). Previous research has documented the negative consequences of mobile phone addiction on college students’ physical and mental health, as well as their academic performance and social functioning (Alhassan et al., 2018). For instance, excessive mobile phone use has been associated with poor sleep quality, decreased physical activity, and increased risk of depression and anxiety (Demirci et al., 2015; Lepp et al., 2013). Moreover, mobile phone addiction can interfere with face-to-face communication and lead to social isolation and loneliness (Bian and Leung, 2015; Sarman and Çiftci, 2024; Erdem and Sezer Efe, 2022).

Given the adverse impacts of mobile phone addiction, it is crucial to identify protective factors that may help prevent or mitigate this problem. One potential protective factor is physical activity, which has been shown to have numerous physical and psychological benefits (Warburton et al., 2006). Our theoretical framework integrates two complementary perspectives to explain how physical activity may reduce mobile phone addiction. First, according to the self-determination theory (Ryan and Deci, 2000), engaging in physical activity can satisfy individuals’ basic psychological needs for autonomy, competence, and relatedness, thereby promoting their well-being and reducing the risk of addictive behaviors. When these needs are fulfilled through physical activity, individuals may feel less compelled to seek need satisfaction through excessive mobile phone use. Second, the Displacement Hypothesis (Kushlev and Leitao, 2020) suggests that time spent on physical activity naturally reduces the time available for mobile phone use. This time displacement creates both behavioral competition (competing for limited time resources) and psychological competition (competing for attention and interest), potentially breaking habitual patterns of excessive mobile phone use. These theoretical perspectives provide a comprehensive framework for understanding the protective role of physical activity against mobile phone addiction. Indeed, empirical evidence has suggested that physical activity is negatively associated with various types of addictive behaviors, such as substance abuse and internet addiction (Wang et al., 2014; Park, 2014). However, the relationship between physical activity and mobile phone addiction among college students has received less attention. The few existing studies on this topic have yielded mixed results (Kim et al., 2015; Gao et al., 2016), highlighting the need for further investigation.

Furthermore, it remains unclear how physical activity may influence mobile phone addiction. One possible mechanism is through the reduction of social anxiety and loneliness, two common psychological problems among college students that have been linked to mobile phone addiction (Enez Darcin et al., 2016; Bian and Leung, 2015). Social anxiety refers to the persistent fear of social or performance situations in which embarrassment or humiliation may occur (American Psychiatric Association, 2013). Individuals with high levels of social anxiety may rely on mobile phones as a “safety blanket” to avoid face-to-face interactions and alleviate their anxiety (King et al., 2013). Loneliness, on the other hand, is defined as the subjective feeling of being alone, disconnected, or separated from others (Peplau and Perlman, 1982). Lonely individuals may turn to mobile phones as a substitute for real-life social connections and to cope with their negative emotions (Sar, 2013).

Physical activity has been found to be effective in reducing social anxiety and loneliness. For example, engaging in regular exercise has been shown to decrease social anxiety symptoms and improve social skills and self-esteem (Li et al., 2024; Deng and Wang, 2024; Wu et al., 2025). Similarly, participation in physical activity can provide opportunities for social interaction and support, thus alleviating feelings of loneliness (Lindsay Smith et al., 2017; Ahn et al., 2024; Lippke et al., 2021). Based on these findings, it is plausible that physical activity may protect against mobile phone addiction by reducing social anxiety and loneliness. The current study aims to examine the relationship between physical activity and mobile phone addiction among Chinese college students and to investigate the potential mediating roles of social anxiety and loneliness in this relationship. Based on the theoretical frameworks and empirical evidence discussed above, we propose the following hypotheses:

*H1:* Physical activity will be negatively associated with mobile phone addiction among Chinese college students.

*H2a:* Social anxiety will mediate the relationship between physical activity and mobile phone addiction.

*H2b:* Loneliness will mediate the relationship between physical activity and mobile phone addiction.

**Figure 1:**
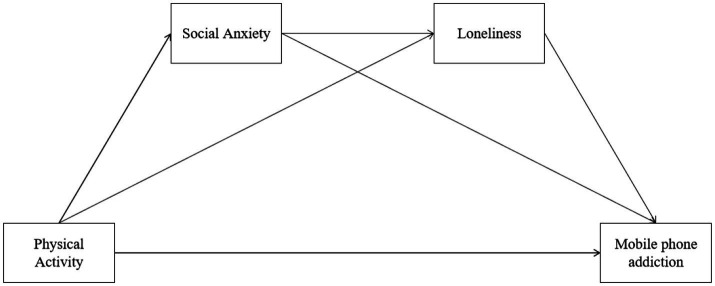
Hypothesized relationships among physical activity, social anxiety, loneliness, and mobile phone addiction.

*H3:* Social anxiety and loneliness will play a chain and parallel mediating role in the relationship between physical activity and mobile phone addiction (Figure 1).

By testing these hypotheses, this study contributes to the literature in several ways. First, it provides new evidence on the association between physical activity and mobile phone addiction among college students, a population that is particularly susceptible to this problem. Second, it sheds light on the underlying mechanisms through which physical activity may influence mobile phone addiction, namely by reducing social anxiety and loneliness. Finally, the findings of this study may inform the development of interventions that promote physical activity as a means of preventing and treating mobile phone addiction among college students. To the best of our knowledge, this is the first study to examine the chain mediating roles of social anxiety and loneliness in the relationship between physical activity and mobile phone addiction. While previous studies have investigated these variables separately or as single mediators, none has explored the chain mechanism through which physical activity influences social anxiety, which in turn affects loneliness, ultimately impacting mobile phone addiction. This integrated approach provides a more comprehensive understanding of the psychological pathways linking physical activity to reduced mobile phone addiction.

## 2 Materials and methods

### 2.1 Participants and procedure

A cross-sectional survey was conducted among college students in a large university in Shandong Province, China. The study was approved by the Ethics Committee of Shandong University (No. 2021–1-114) and followed the guidelines of the Declaration of Helsinki. A convenience sampling method was used to recruit participants. The investigators were trained to guide the survey process and answer participants’ questions. Informed consent was obtained from all participants before they proceeded to the survey. Participants were assured of the anonymity and confidentiality of their responses. To ensure adequate statistical power for our analyses, we conducted an *a priori* power analysis using G*Power 3.1. For multiple regression analyses with three predictors, medium effect size (*f*^2^ = 0.15), *α* = 0.05, and power = 0.95, the minimum required sample size was 119 (Faul et al., 2009). For mediation analyses, Fritz and MacKinnon (2007) recommend a minimum sample size of 148 to detect medium effect sizes in the paths of mediation models. Oversampling was undertaken to mitigate the potential exclusion of data due to low compliance.

A total of 300 questionnaires were distributed, and 268 valid questionnaires were collected, yielding a response rate of 89.3%. The sample consisted of 168 males (62.7%) and 100 females (37.3%), with a mean age of 20.36 years (SD = 1.73). Among the participants, 195 (72.8%) were from urban areas, and 73 (27.2%) were from rural areas. The distribution of participants across academic years was as follows: 50 freshmen (18.7%), 62 sophomores (23.1%), 68 juniors (25.4%), and 88 seniors (32.8%).

### 2.2 Measures

Demographic characteristics were collected through several sociodemographic questions, including age, sex, urban–rural provenance, and grade.

The Physical Activity Rating Scale-3 (PARS-3) (Liang, 1994) was used to assess participants’ physical activity levels. This scale consists of three items measuring the intensity, duration, and frequency of physical activity in the past month. The intensity and frequency items are rated on a 5-point Likert scale, while the duration item is rated on a 4-point Likert scale. The total score is calculated by multiplying the scores of the three items, with higher scores indicating higher levels of physical activity. The PARS-3 has been validated in Chinese college students and has shown good reliability and validity (Feng et al., 2014).

The Social Phobia Inventory (SPIN) (Connor et al., 2000) was used to measure participants’ social anxiety. The SPIN is a 17-item self-report scale that assesses fear, avoidance, and physiological discomfort in social situations. Each item is rated on a 5-point Likert scale ranging from 0 (not at all) to 4 (extremely). The total score ranges from 0 to 68, with higher scores indicating higher levels of social anxiety. The Chinese version of the SPIN has demonstrated good psychometric properties (Gong et al., 2010). In this study, the Cronbach’s alpha coefficient for the SPIN was 0.938.

The UCLA Loneliness Scale (Version 3) (Russell, 1996) was used to assess participants’ loneliness. This scale consists of 20 items, each rated on a 4-point Likert scale ranging from 1 (never) to 4 (always). Nine items are reverse-scored. The total score ranges from 20 to 80, with higher scores indicating higher levels of loneliness. The Chinese version of the UCLA Loneliness Scale has shown good reliability and validity (Jia et al., 2020). In this study, the Cronbach’s alpha coefficient for the scale was 0.721.

The Mobile Phone Addiction Tendency Scale (MPATS) (Xiong et al., 2012) was used to measure participants’ mobile phone addiction. The MPATS is a 16-item self-report scale that assesses four dimensions of mobile phone addiction: withdrawal symptoms, salience, social comfort, and mood changes. Each item is rated on a 5-point Likert scale ranging from 1 (completely disagree) to 5 (completely agree). The total score ranges from 16 to 80, with higher scores indicating higher levels of mobile phone addiction. The MPATS has been validated in Chinese college students and has shown good reliability and validity (Liu et al., 2017). In this study, the Cronbach’s alpha coefficient for the MPATS was 0.895.

### 2.3 Statistical analysis

Data were analyzed using SPSS 22.0. First, Harman’s single-factor test was performed to check for common method bias (Podsakoff et al., 2003). Second, descriptive statistics and Pearson’s correlation coefficients were calculated for the study variables. Third, in order to fully explore the mediating mechanism between physical activity and mobile phone addiction, this study will test multiple mediating models, and the SPSS macro PROCESS (Version 3.3) (Hayes, 2017) was used to test the parallel mediation and chain mediation models. Specifically, we opted for path analysis using the PROCESS macro rather than structural equation modeling (SEM) for several reasons. First, our primary research objective was to test the chain mediation relationships between variables rather than to examine the measurement model of latent constructs. Second, all measures used in this study have been previously validated with established psychometric properties in Chinese populations. Third, the PROCESS macro provides a streamlined approach for testing complex mediation models with bootstrapped confidence intervals, which is particularly suitable for examining indirect effects.

The indirect effects were estimated using a bootstrap procedure with 5,000 resamples. The 95% bias-corrected confidence intervals (CIs) were calculated, and CIs not including zero indicated significant indirect effects. Age, gender, academic year, and urban/rural background were included as covariates in the mediation analyses. Given that these demographic factors may influence college students’ physical activity levels, social anxiety, loneliness, and mobile phone addiction, it is necessary to control for their influence when examining the relationship between the primary interest variables. Specifically, these demographic variables were simultaneously entered as covariates in all equations predicting the mediators (social anxiety and loneliness) and the outcome variable (mobile phone addiction). This approach controls for the potential influences of these demographic characteristics on each pathway in the mediation model.

## 3 Results

### 3.1 Common method bias and descriptive statistics

Harman’s single-factor test was conducted to assess common method bias. The results showed that the first factor explained 22.68% of the variance, which was less than the threshold of 40%, indicating that common method bias was not a significant concern in this study (Podsakoff et al., 2003). Tables 1, 2 presents the descriptive statistics and correlations among the study variables. Physical activity was negatively correlated with social anxiety (*r* = −0.26, *p* < 0.001), loneliness (*r* = −0.25, *p* < 0.001), and mobile phone addiction (*r* = −0.28, *p* < 0.001). Social anxiety was positively correlated with loneliness (*r* = 0.32, *p* < 0.001) and mobile phone addiction (*r* = 0.53, *p* < 0.001). Loneliness was positively correlated with mobile phone addiction (*r* = 0.37, *p* < 0.001).

**Table 1:** Characteristics of athlete participants.

Characteristics	Category	*n*	%
Sex	Male	168	62.7
	Female	100	37.3
Age (years)	17–18 years	43	16.1
	19–20 years	109	40.7
	21–22 years	83	31
	23–25 years	33	12.2
Urban–rural provenance, *n* (%)	Rural	73	27.2
	Urban	195	72.8
Grade, *n* (%)	Freshman	50	18.7
	Sophomore	62	23.1
	Junior	68	25.4
	Senior	88	32.8

Data were described as *n* (%). Average age = 20.36 years (SD = 1.73).

**Table 2:** Descriptive statistics and bivariate correlations (*n* = 268).

Variables	*M*	SD	1	2	3
Physical activity	26.53	22.63	1		
Social anxiety	2.53	0.85	−0.263^***^	1	
Loneliness	2.48	0.16	−0.252^***^	0.324^***^	1
Mobile phone addiction	3.10	0.70	−0.279^***^	0.534^***^	0.370^***^

***p < 0.001. *M*, mean; SD, standard deviation.

### 3.2 Parallel mediation analysis

The parallel mediation analyses were conducted using the SPSS macro PROCESS (Version 3.3) [31]. Model 4 was used to test the mediating effects of social anxiety and loneliness separately, and this parallel model includes two separate simple mediation pathways (physical activity → social anxiety → mobile phone addiction; physical activity → loneliness → mobile phone addiction). Age, gender, academic year, and urban/rural background were included as covariates in all mediation analyses.

**Figure 2:**
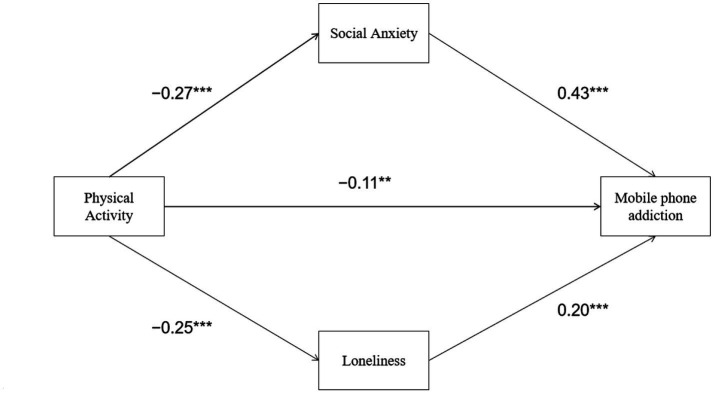
The mediating effect of social anxiety and loneliness in a parallel model.

The results (Tables 3, 4; Figure 2) showed that physical activity had a significant negative effect on social anxiety (*β* = −0.27, *p* < 0.001), and social anxiety had a significant positive effect on mobile phone addiction (*β* = 0.43, *p* < 0.001). The direct effect of physical activity on mobile phone addiction was significant (*β* = −0.11, *p* < 0.05). The indirect effect of physical activity on mobile phone addiction through social anxiety was significant (*β* = −0.12, 95% CI [−0.18, −0.06]), indicating that social anxiety partially mediated the relationship between physical activity and mobile phone addiction.

**Table 3:** Multiple linear regression results for testing the mediating role of social anxiety and loneliness in the relationship between physical activity and mobile phone addiction after controlling for demographic variables (*n* = 268).

Predictor variable	Outcome variable	*R*	*R* ^2^	*F*	*β*	*t*	Boot LLCI	Boot ULCI
Equation 1								
Physical activity	Social anxiety	0.35	0.12	7.24	−0.27	−4.53^***^	−0.39	−0.15
Equation 2								
Physical activity	Loneliness	0.26	0.07	3.69	−0.25	−4.09^***^	−0.37	−0.13
Equation 3								
Physical activity	Mobile phone addiction	0.59	0.35	19.93	−0.11	−2.07^**^	−0.22	−0.01
Social anxiety					0.43	7.77^***^	0.32	0.54
Loneliness					0.20	3.64^***^	0.09	0.30

All variables used in this table were standardized. Boot LLCI and boot ULCL are, respectively, the lower 95% confidence interval and upper 95% confidence interval, calculated by bias-corrected percentile bootstrap, for testing indirect effects. 95% confidence intervals do not overlap with zero. R (multiple correlation coefficient) represents the strength of the relationship between the predictors and the outcome variable; R^2^ (coefficient of determination) indicates the proportion of variance in the outcome variable explained by the predictors; F (F-statistic) tests the overall significance of the regression model; β (standardized regression coefficient) reflects the change in the outcome variable associated with one standard deviation change in the predictor, while controlling for other variables; and t (t-statistic) tests the significance of individual regression coefficients. ***p < 0.001; **p < 0.05.

**Table 4:** The comparison of the mediating effect of social anxiety and loneliness after controlling for demographic variables (*n* = 268).

Pathway	Effect	Boot SE	Boot LLCI	Boot ULCI	Ratio of indirect to total effect	Ratio of indirect to direct effect
Total effect	−0.28	0.06	−0.40	−0.16	–	–
Direct effect	−0.11	0.05	−0.22	−0.01	–	–
Total indirect effect	−0.17	0.04	−0.24	−0.10	59.68%	146.71%
Mediating effect of social anxiety	−0.12	0.03	−0.19	−0.06	41.39%	102.11%
Mediating effect of loneliness	−0.05	0.01	−0.09	−0.01	18.08%	44.59%

The mediating effect of social anxiety was physical activity → social anxiety → mobile phone addiction. The mediating effect of loneliness was physical activity → loneliness → mobile phone addiction. Boot SE, boot LLCI, and boot ULCL are the estimated standard errors, lower bound of the 95% confidence interval, and the upper bound of the 95% confidence interval by the bias-corrected percentile bootstrap method for testing indirect effects. 95% confidence intervals do not overlap with zero.

The results showed that physical activity had a significant negative effect on loneliness (*β* = −0.25, *p* < 0.001), and loneliness had a significant positive effect on mobile phone addiction (*β* = 0.20, *p* < 0.001). The direct effect of physical activity on mobile phone addiction was significant (*β* = −0.11, *p* < 0.05). The indirect effect of physical activity on mobile phone addiction through loneliness was significant [*β* = −0.05, 95% CI (−0.09, −0.01)], indicating that loneliness partially mediated the relationship between physical activity and mobile phone addiction.

### 3.3 Chain mediating effect of social anxiety and loneliness

**Figure 3:**
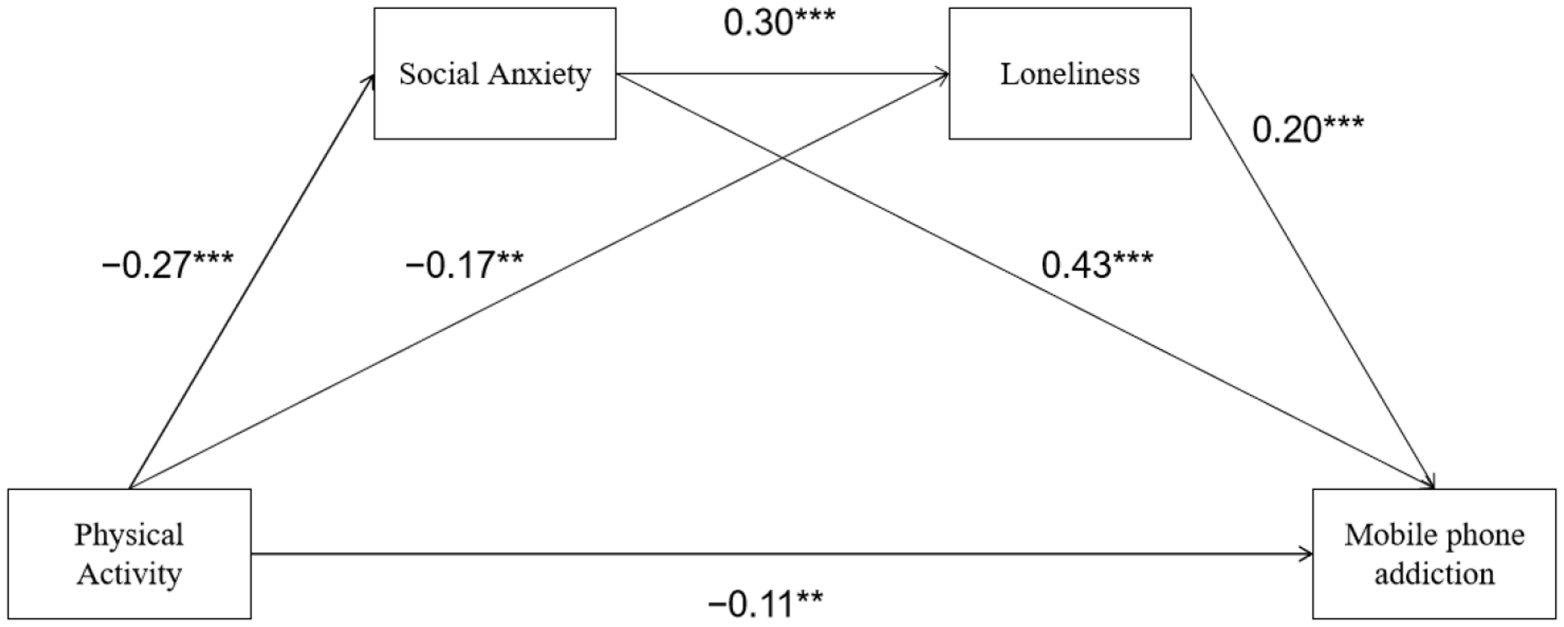
The chain mediating effect of social anxiety and loneliness.

In addition to testing the hypothesized, this study also examined a chain mediation model. This approach allows us to assess the relative contribution of each mediating pathway while controlling for the others, providing a more nuanced understanding of the mechanisms linking physical activity to mobile phone addiction. The results (Tables 5, 6; Figure 3) showed that physical activity had a significant negative effect on social anxiety (*β* = −0.27, *p* < 0.001), social anxiety had a significant positive effect on loneliness (*β* = 0.30, *p* < 0.001), and loneliness had a significant positive effect on mobile phone addiction (*β* = 0.20, *p* < 0.001). The direct effect of physical activity on mobile phone addiction was significant (*β* = −0.11, *p* < 0.05). The indirect effect of physical activity on mobile phone addiction through social anxiety and loneliness was significant [*β* = −0.02, 95% CI (−0.03, −0.01)], indicating that social anxiety and loneliness had a significant chain mediating effect on the relationship between physical activity and mobile phone addiction. The total indirect effect of physical activity on mobile phone addiction through social anxiety and loneliness was significant [*β* = −0.17, 95% CI (−0.24, −0.10)], accounting for 59.68% of the total effect. Specifically, the indirect effect through social anxiety accounted for 41.97% of the total effect, the indirect effect through loneliness accounted for 12.09% of the total effect, and the chain mediating effect through social anxiety and loneliness accounted for 5.63% of the total effect. The results showed that the direct effect of physical activity on loneliness was stronger in the parallel model (*β* = −0.25) than in the chain model (*β* = −0.17), suggesting that the parallel model may have overlooked the mediating role of social anxiety on loneliness. The chain model provides a more comprehensive mechanistic explanation, suggesting that social anxiety may be a precursor variable for loneliness (Figure 3).

**Table 5:** Multiple linear regression results for testing the mediating role of social anxiety in the relationship between physical activity and loneliness, and their combined effects on mobile phone addiction after controlling for demographic variables (*n* = 268).

Predictor variable	Outcome variable	*R*	*R* ^2^	*F*	*β*	*t*	Boot LLCI	Boot ULCI
Equation 1								
Physical activity	Social anxiety	0.35	0.12	7.24	−0.27	−4.53^***^	−0.39	−0.15
Equation 2								
Physical activity	Loneliness	0.38	0.14	7.26	−0.17	−2.80^**^	−0.29	−0.05
Social anxiety					0.30	4.85^***^	0.18	0.42
Equation 3								
Physical activity	Mobile phone addiction	0.59	0.35	19.93	−0.11	−2.07^**^	−0.22	−0.01
Social anxiety					0.43	7.77^***^	0.32	0.54
Loneliness					0.20	3.64^***^	0.09	0.30

All variables used in this table were standardized. Boot LLCI and boot ULCL are respectively the lower 95% confidence interval and upper 95% confidence interval, calculated by bias-corrected percentile bootstrap, for testing indirect effects. 95% confidence intervals do not overlap with zero. ***p < 0.001; **p < 0.05.

**Table 6:** Indirect effect of social anxiety and loneliness after controlling for demographic variables (*n* = 268).

	Effect	Boot SE	Boot LLCI	Boot ULCI	Ratio of indirect to total effect	Ratio of indirect to direct effect
Total indirect effect	−0.17	0.03	−0.24	−0.10	59.68%	148%
Indirect effect 1	−0.12	0.03	−0.18	−0.06	41.97%	104%
Indirect effect 2	−0.03	0.01	−0.06	−0.01	12.09%	29%
Indirect effect 3	−0.02	0.01	−0.03	−0.01	5.63%	13%

Indirect effect 1 is physical activity → social anxiety → tendency of mobile phone addiction. Indirect effect 2 is physical activity → loneliness → mobile phone addiction tendency. Indirect effect 3 is physical activity → social anxiety → loneliness → mobile phone addiction tendency. Boot SE, boot LLCI, and boot ULCL are estimated standard errors, lower limits of 95% confidence intervals, and upper limits of 95% confidence intervals by bias-corrected percentile bootstrap method for testing indirect effects. 95% confidence intervals do not overlap with zero.

## 4 Discussion

### 4.1 Summary of findings

The present study found that physical activity was significantly negatively associated with mobile phone addiction, while both social anxiety and loneliness independently mediated this relationship. Most importantly, this study found a significant chain mediating effect through social anxiety and loneliness. This chain mechanism provides new insights into the complex psychological pathways linking physical activity to reduced mobile phone addiction.

### 4.2 Physical activity and mobile phone addiction

The findings align with previous research demonstrating the protective effects of physical activity against various addictive behaviors (Kim et al., 2015; Lepp et al., 2013), supporting hypothesis 1. Recent research has confirmed that physical activity remains a significant protective factor against mobile phone addiction. For example, the findings are particularly consistent with a recent study Wang (2025), which found that physical activity can reduce mobile phone addiction in university students and identified psychological mediators in this relationship. This research advances understanding by identifying specific psychological mechanisms underlying this relationship. This relationship can be explained by the displacement hypothesis, which suggests that the time spent on physical activity may reduce the time available for mobile phone use, thus decreasing the risk of mobile phone addiction (Xu et al., 2018; Kushlev and Leitao, 2020). Moreover, engaging in physical activity can provide individuals with a sense of achievement, improve their mood, and reduce stress (Mandolesi et al., 2018), which may reduce their reliance on mobile phones as a coping strategy for negative emotions.

### 4.3 Mediating role of social anxiety

The mediating role of social anxiety in the relationship between physical activity and mobile phone addiction is a novel finding of this study, supporting hypotheses 2a. Previous studies have shown that physical activity can alleviate social anxiety symptoms (Ashdown-Franks et al., 2020), and social anxiety is positively associated with mobile phone addiction (Enez Darcin et al., 2016). The identification of social anxiety as a primary mediator complements Gan and Jiang (2024) findings that physical activity reduces social anxiety symptoms among college students, though they explored different moderators (self-esteem) and mediators (body image). Specifically, physical activity may provide opportunities for positive social interactions and improve social skills (Eime et al., 2013), which can help reduce social anxiety. Individuals with lower levels of social anxiety are less likely to use mobile phones as a means to avoid face-to-face interactions and cope with social distress, thus reducing the risk of mobile phone addiction.

### 4.4 Mediating role of loneliness

The mediating role of loneliness found in this study is consistent with previous findings that have shown a negative association between physical activity and loneliness (Lindsay Smith et al., 2017) and a positive association between loneliness and mobile phone addiction (Sar, 2013), supporting hypotheses 2a. The mediating role of loneliness identified in the present study builds on the findings of previous scholars on the impact of physical activity on social isolation, but provides new evidence for loneliness as a specific mechanism in the context of mobile phone addiction. A recent review conducted by Ahn et al. (2024) highlights that interventions targeting physical activity can also alleviate loneliness. This study further demonstrates that physical activity can reduce mobile phone addiction by alleviating feelings of loneliness. Engaging in physical activity, especially group-based activities, can provide opportunities for social connections and support, which can help satisfy the need for belongingness and reduce loneliness (Haugen et al., 2013). Individuals who feel less lonely are less likely to rely on mobile phones for social interaction and emotional comfort, thus decreasing the risk of mobile phone addiction.

### 4.5 Chain mediating effect of social anxiety and loneliness

While earlier studies established the direct relationship between physical activity and mobile phone addiction, this study advances beyond these works by identifying the sequential chain through which physical activity influences mobile phone addiction. This chain mechanism of physical activity → social anxiety → loneliness → mobile phone addiction has not been previously documented in the mobile phone addiction literature, though similar chain mediation processes have been observed in recent studies (Wang, 2025), supporting hypotheses 3. The chain mediating effect of social anxiety and loneliness found in this study provides a more comprehensive understanding of the mechanisms underlying the relationship between physical activity and mobile phone addiction. This finding suggests that physical activity can reduce social anxiety, which in turn can reduce loneliness, ultimately leading to a lower risk of mobile phone addiction. Zhao et al. (2022) revealed that psychological resilience and perceived stress have a chain mediating effect between physical exercise and mobile phone addiction in college students, similar to this chain mediation model involving social anxiety and loneliness. Previous studies have shown that social anxiety and loneliness are interrelated, with social anxiety being a risk factor for loneliness (Maes et al., 2019). The connection between social anxiety reduction and decreased loneliness demonstrated in current chain mediation model extends Maes et al.'s (2019) work on the relationship between these variables, providing new evidence for their sequential rather than merely correlational relationship. Individuals with high levels of social anxiety may have difficulties in forming and maintaining social relationships, leading to increased feelings of loneliness (Lim et al., 2016). By reducing social anxiety through physical activity, individuals may be more likely to engage in social interactions and build social connections, thus reducing loneliness and the reliance on mobile phones for social interaction. The negative association between physical activity and mobile phone addiction can be understood through complementary theoretical frameworks. For example, these findings may be explained by self-determination theory (Ryan and Deci, 2000), which states that the satisfaction of basic psychological needs may specifically reduce social anxiety and subsequent loneliness because individuals build greater social confidence and connection through physical activity. When college students engage in regular physical activity, they experience a sense of accomplishment and social connection that reduces their dependency on mobile phones for psychological satisfaction. The displacement hypothesis further explains why this relationship exists - time devoted to physical activity naturally limits opportunities for phone use, creating both temporal and psychological competition with problematic phone behaviors (Kushlev and Leitao, 2020). Physical activity likely provides a non-threatening context for social interaction, where focus remains on the activity rather than social evaluation, thus allowing individuals to develop social confidence before deeper connections. As social anxiety decreases, students become more willing to engage in meaningful social relationships, naturally reducing loneliness. This decreased loneliness then diminishes the need to use mobile phones as a substitute for real-world social connections. This mechanism extends beyond simple time displacement, suggesting that physical activity initiates a cascade of psychological benefits that collectively protect against mobile phone addiction.

### 4.6 Theoretical and practical implications

The study makes a unique contribution to the literature by revealing, for the first time, the chain mediating effect of social anxiety and loneliness in the relationship between physical activity and mobile phone addiction. Unlike previous research that has focused on isolated relationships or single mediating variables, this study provides a more nuanced understanding of the sequential psychological mechanisms through which physical activity may protect against mobile phone addiction. The findings of this study also have important practical implications for preventing and reducing mobile phone addiction among college students. First, for college students, physical activity should be realized as an effective protective factor to reduce mobile phone addiction, and regular physical activity should be integrated into daily life. Such as actively participating in group sports activities that provide opportunities for social connection and support, setting time limits on cell phone use, and consciously replacing excessive cell phone use with face-to-face social interactions and physical activities. Second, for mental health practitioners working with college students, the findings suggest a multi-component approach to addressing mobile phone addiction that targets each element in the mediation chain. This could include prescribing regular physical activity as part of treatment protocols, incorporating exposure therapy elements to reduce social anxiety, and facilitating meaningful social connections to reduce loneliness. For example, social skills training, cognitive-behavioral therapy, and mindfulness-based interventions have been shown to be effective in reducing social anxiety and loneliness (Kampmann et al., 2018; Masi et al., 2011). These interventions can be delivered through individual or group therapy sessions, as well as through self-help programs using mobile apps or online platforms. Third, for policymakers, the results suggest the value of investing in campus recreational facilities and creating policies that encourage regular physical activity, potentially including physical activity requirements in university curricula and developing campus spaces that facilitate both exercise and social interaction.

### 4.7 Limitations and future research directions

Despite the strengths of this study, there are also several limitations that should be acknowledged. First, the cross-sectional design of this study precludes causal inferences about the relationships among physical activity, social anxiety, loneliness, and mobile phone addiction. Future studies should use a longitudinal design to establish causality. Second, the use of self-report measures may be subject to social desirability bias and recall bias. Future studies could integrate neuroscientific approaches (e.g., fMRI) to investigate the neural mechanisms of mobile phone addiction. Third, one limitation of this study is the relatively small sample size, which may affect the generalizability of the findings. Future research should replicate this study with a larger and more diverse sample, further explore its applicability in different populations and examine the long-term effects of effective intervention measures. Moreover, future studies should consider employing structural equation modeling to simultaneously examine both measurement and structural models, which would provide a more comprehensive understanding of the relationships among these constructs. Finally, this study did not assess potential moderators such as personality traits, motivations for phone use, or types of physical activity, which might influence the strength or direction of the observed relationships.

## 5 Conclusion

In conclusion, this study provides evidence for the protective role of physical activity against mobile phone addiction among Chinese college students and reveals the mediating roles of social anxiety and loneliness in this relationship. The findings highlight the importance of promoting physical activity and reducing social anxiety and loneliness in preventing and reducing mobile phone addiction among college students.

## Data Availability

The raw data supporting the conclusions of this article will be made available by the authors, without undue reservation.
